# Engineering Extracellular Vesicles Derived from 3D Cultivation of BMSCs Enriched with HGF Ameliorate Sepsis‐Induced Lung Epithelial Barrier Damage

**DOI:** 10.1002/advs.202500637

**Published:** 2025-03-05

**Authors:** Yong Chen, Feihong Lin, Tong Zhang, Zhuoran Xiao, Yuanli Chen, Dongsheng Hua, Yu Wang, Juan Wei, Tian Jin, Xin Lv

**Affiliations:** ^1^ Department of Anesthesiology Shanghai Pulmonary Hospital School of Medicine Tongji University Shanghai 200433 China; ^2^ Shanghai Institute of Acupuncture and Anesthesia Shanghai 200433 China; ^3^ Department of Anesthesiology The First Affiliated Hospital of Wenzhou Medical University Wenzhou 325000 China

**Keywords:** acute lung injury, extracellular vesicles, mesenchymal stem cell, sepsis, 3D culture

## Abstract

Sepsis is a critical condition with high mortality, often leading to acute lung injury (ALI) due to uncontrolled inflammatory responses and alveolar epithelial damage. Extracellular vesicles (EVs), particularly mesenchymal stem cell‐derived EVs, have shown therapeutic potential in sepsis‐related organ dysfunction by transferring RNAs and proteins. However, their clinical use is limited by low efficacy and yield. To address this, 3D‐cultured MSCs (3D‐MSCs) are generated using MicroTissues 3D Petri Dish. These 3D‐MSCs demonstrate improved protection and proliferation of MLE‐12 cells in vitro. Mechanistic studies are conducted to explore the enhanced protective effects of 3D‐MSCs derived EVs (3D‐EVs) in a septic‐ALI model. Proteomic and molecular analyses of 3D‐EVs revealed that they are enriched in hepatocyte growth factor (HGF). HGF helps maintain the barrier function of damaged alveolar epithelium through the PI3K‐AKT signaling pathway. Overall, 3D‐EVs effectively ameliorate sepsis‐induced ALI and enhance prognosis by enriching and delivering HGF, suggesting that their application represents a promising treatment strategy for septic ALI.

## Graphical abstract

3D culture of MSCs upregulates gene expression of matrix metalloproteinases (MMPs), growth factors (GFs), and increases the production of extracellular vesicles (EVs). Proteomic analysis reveals 642 upregulated proteins in 3D‐EVs, including HGF. These 3D‐EVs, enriched with HGF, protect lung epithelial cells from apoptosis and barrier damage in septic ALI via the PI3K/AKT pathway. Figure created with Biorender.com.

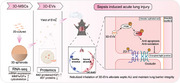

## 1 Introduction

Sepsis is a common and severe disease with high mortality in critically ill patients, imposing a heavy economic burden.^[^ ^1^ ^]^ Over the years, the incidence has increased gradually due to population aging. Among the multiple organ dysfunctions that sepsis can cause, the lung is often the first target of damage because of its susceptibility, developing into acute lung injury (ALI) and acute respiratory distress syndrome (ARDS). The pathogenesis of ALI/ARDS is characterized by diffuse alveolar damage, which encompasses injury to pulmonary capillary endothelial cells and alveolar epithelial cells, along with interstitial and alveolar edema.^[^ ^2^ ^]^ Despite the growing concern surrounding sepsis‐induced lung injury, no effective pharmacological therapy has been identified.

Given the lack of effective pharmacological therapies for sepsis‐induced lung injury, mesenchymal stromal cells (MSCs) have emerged as a promising therapeutic option. Bone marrow MSCs (BMSCs) represent a population of multipotent stem cells endowed with self‐renewal capabilities.^[^ ^3^ ^]^ The administration of MSCs has been shown to mitigate alveolar‐capillary injury and reduce mortality in pre‐clinical models of ALI, owing to their multifaceted therapeutic properties, including anti‐inflammatory, immunomodulatory, regenerative, proangiogenic, and antimicrobial effects.^[^ ^4^ ^]^ A growing body of evidence suggests that the primary mode of MSC action is mediated through their secretome, particularly extracellular vesicles (EVs).^[^ ^5^ ^]^ EVs secreted by MSCs (MSC‐EVs) mirror the cells' inherent activity by delivering functional biomolecules, such as lipids, cytosolic proteins, and biologically active RNAs, to sites of tissue damage.^[^ ^6^ ^]^ Experimental evidence has demonstrated that MSCs can transfer EVs to target cells, effectively promoting the recovery of epithelial and endothelial tissues and facilitating microbial clearance and alveolar fluid reabsorption.^[^ ^7^ ^]^ Our previous research, along with that of others, has positioned MSC‐EVs as a promising avenue for cell‐free therapy in ALI.^[^ ^8^ ^]^ Studies have also shown that the paracrine properties of MSCs can be modulated through various preconditioning methods and techniques.^[^ ^9^ ^]^ For instance, the shift from a monolayer to a nonadhesive, 3D culture environment has been found to significantly alter the composition of the secretome, potentially augmenting its therapeutic efficacy.^[^ ^10^ ^]^ Dynamic 3D culture of MSC‐EVs has been shown to rejuvenate senescent stem cells and enhance their immunomodulatory potential.^[^ ^11^ ^]^ Moreover, exosomes produced from 3D‐cultured MSCs exhibit increased yield, albeit to varying degrees.^[^ ^12^ ^]^ Collectively, these findings underscore the potential of 3D cell cultures to overcome the limitations hindering the broad application of EVs.

In this study, we harnessed a non‐adhesive 3D aggregation culture system to cultivate MSCs and evaluated the therapeutic efficacy of 3D‐MSCs on injured lung epithelial cells in vitro. To elucidate the underlying mechanisms, we conducted RNA‐sequencing analysis, offering a comprehensive view of transcriptome alterations following 3D culture generation, with a particular emphasis on cellular remodeling and EVs generation pathways. Concurrently, it has been established that the paracrine factors derived from MSC spheroids can enhance tissue repair and immunomodulatory effects.^[^ ^13^ ^]^ Consequently, we purified and characterized the 3D‐cultured MSC‐derived EVs in their entirety. Subsequently, we evaluated the roles of 2D‐EVs and 3D‐EVs in preserving alveolar epithelial barrier integrity using a sepsis‐induced ALI mouse model. Our proteomic analyses revealed that the therapeutic mechanisms of these EVs involve, at least in part, the transfer of enriched hepatocyte growth factor (HGF). Therefore, our data provide evidence that 3D‐EVs may exert a significant impact on the reparative processes of lung epithelial tissue in sepsis.

## 2 Experimental Section

### 2.1 Culture of MSCs from Bone Marrow

The BMSCs were obtained from Cyagen Biosciences (Guangzhou, China) and cultured in DMEM/F12 (Gibco) containing 10% fetal bovine serum (Gibco) and 1% penicillin/streptomycin (Gibco). The cells were placed in 100 mm cell culture dishes (Corning, NY, USA) and maintained at a temperature of 37 °C with 5% CO2 and 90% humidity. The growth medium was replaced every three days, and cell passage was conducted once the cells reached 90% confluence. After removing the culture medium, the cells that adhered to the surface were rinsed two times using phosphate‐buffered saline (PBS). Subsequently, these cells were collected by exposing them to 1 mL of 0.25% trypsin and 1 mm ethylenediaminetetraacetic acid solution for a duration of 2 min at a temperature of 37 °C. This was followed by adding 5 mL of complete medium to counteract the effects of trypsin. Ultimately, the cells were reintroduced into a fresh medium at a ratio of 1:3 dilution. For all subsequent experiments, the utilization of BMSCs was limited to passages 3–5. The confirmation of BMSCs' identity involved flow cytometric detection of common MSC markers. This entailed the use of FITC‐conjugated monoclonal antibody against CD45, CD49e, and CD90, PE‐conjugated monoclonal antibody against CD29, along with their respective isotype controls (eBioscience, San Diego, CA, USA).

### 2.2 Preparation of Agarose Micro Multi‐Well Dishes and 3D MSCs Spheroids

**Figure 1:**
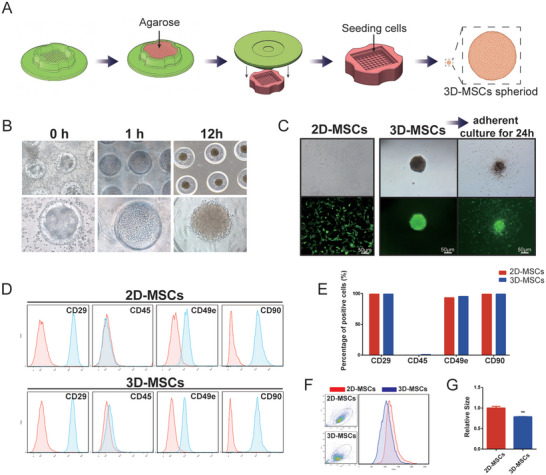
Characterization of 2D and 3D‐MSCs. A) Schematic diagrams of the establishment of 3D culture systems. B) Formation of MSC spheroids in 3D culture dishes. C) Calcein‐AM staining analysis of 2D, 3D‐MSCs and readhered cultured 3D‐MSCs. D,E) Flow cytometry analysis of surface markers of 2D and 3D‐MSCs. F,G) FSC/SSC analysis of 2D and 3D‐MSCs. FSC (Scattered light) reflects the relative size of cells. Mean ± SE, *n* = 4, ^**^ *P* < 0.01.

81‐wells rubber micro‐molds (**Figure** **1**A) with a diameter of 400 µm were procured from Micro Tissues Inc (Providence, RI, USA). The molds were subjected to autoclave sterilization and subsequently dried in an ultraviolet irradiation setting for a duration of 1 h. Two percent (g/mL) agarose powder (Sigma) was mixed with DMEM/F12 and autoclave sterilized. While the mixture was still in liquid form, 500 µL of it was carefully pipetted into each mold. Following solidification, the micro‐well plates were extracted employing a sterilized shovel. The agarose dishes were placed in 6‐well culture Petri dishes with 2 mL DMEM/F12 containing 1% P/S and incubated at 37 °C with 5% CO2 for 24 h. Prior to adding MSCs to the micro‐well plates, the medium was removed from both the culture dishes and the micro‐well plates. Next, 200 µL of cell suspension, containing 2 × 10^5^ cells mL^−1^, was carefully added to each well of the micro‐well plates. Then, 2 mL of DMEM/F12 with 10% exosome‐depleted FBS and 1% P/S was added to the 12‐well culture Petri dishes (Corning, NY, USA). The culture dishes were incubated in a 37 °C incubator of 5% CO2 for 48 h and the medium was collected for EVs isolation.

### 2.3 Treatment and detection of MLE‐12 Cells

The murine lung epithelial cell line MLE‐12, procured from the American Type Culture Collection (Manassas, VA, USA), was cultured in Dulbecco's Modified Eagle Medium (DMEM; Gibco) supplemented with 10% fetal bovine serum (FBS; Gibco) and 1% penicillin/streptomycin (Gibco). The cells were seeded to a 6‐well culture plate at a density of 10^6^ cells per well and cultured for 24 h. Subsequently, the cells were challenged with lipopolysaccharide (LPS; 1 µg mL^−1^; Sigma, USA) and tumor necrosis factor‐alpha (TNF‐α; 10 ng mL^−1^; Sigma, USA) for 24 h to induce a model of pulmonary injury in vitro. Concurrently, the cells were co‐cultured with 1 × 10^5^ stem cells or treated with 10 µg mL^−1^ of EVs to assess their potential protective effects. After a 24‐h incubation, the cells were harvested for subsequent analysis.

The quantification of early and late apoptotic events was performed using the Annexin V‐FITC/PI Apoptosis Detection kit (BD Biosciences). After a preliminary wash, cells were resuspended in annexin V‐binding buffer and subjected to staining with FITC‐conjugated Annexin V and propidium iodide (PI) for a duration of 15 min. The subsequent analysis was conducted employing a flow cytometer (BD FACSAria, Rockville, MD) to distinguish apoptotic populations.

Reactive oxygen species (ROS) levels were determined using the DCFH‐DA assay kit (Solarbio, China), adhering to the manufacturer's protocol. Cells, pre‐treated as previously described, were incubated with DCFH‐DA in a dark chamber at 37 °C for 20 min, after which the fluorescence intensity was assessed using a flow cytometer to indicate ROS production.

Mitochondrial membrane potential (MMP) was evaluated using the JC‐1 assay kit, following the manufacturer's guidelines. Cells, pre‐treated as outlined, were incubated with JC‐1 (5 m m) for 30 min, and the resulting fluorescence emissions were analyzed by flow cytometry. The MMP was calculated based on the ratio of red to green fluorescence emissions.

Cell proliferation was assessed using the EdU Cell Proliferation Kit and Cell Cycle Analysis Kit (Beyotime, China), in accordance with the manufacturer's instructions. For the scratch wound assay, MLE‐12 cells were cultured to confluence in 12‐well plates at a seeding density of 1 × 10^5^ cells per well. A sterilized 1 mL pipette tip was utilized to create a scratch in the cell monolayer, which was then treated with MSCs or EVs, as detailed in the preceding sections. After a 24 h incubation, microscopy was employed to observe cellular migration and to quantify the remaining open wound area.

### 2.4 Isolation and Identification of EVs

After a 48‐hour incubation period, the culture medium was collected and processed through a series of centrifugation steps to remove floating cells, debris, and cellular remnants. Initial centrifugation was performed at 300 g for 10 min, followed by 2000 g for 10 min, and finally 10000 g for 30 min. The supernatant was then filtered through a 0.22‐µm pore size filter to ensure sterility and purity. Ultracentrifugation at 100000 g for 90 min was subsequently employed to pellet EVs, which were once washed with PBS. The purified EVs were resuspended in PBS and stored at −80 °C for further analysis.

Concurrently, the size distribution and concentration of the EVs were determined using nanoparticle tracking analysis (NTA) with the Flow NanoAnalyzer (NanoFCM, China). The morphology of the EVs was examined under transmission electron microscopy (TEM, Japan) to provide insights into their structural characteristics. To semi‐quantify the EV concentration, the protein content of EVs derived from the culture medium of both 2D‐MSCs and 3D‐MSCs was measured using the bicinchoninic acid (BCA) protein assay kit (Beyotime, China), as previously described. The presence of EV protein markers, including HSP70, Tsg101, and CD81, was confirmed by Western blot analysis, with specific antibody details provided in Table S1 (Supporting Information).

### 2.5 Quantitative Real‐Time Polymerase Chain Reaction (qPCR)

The Trizol reagent (Invitro gen, Calif) was used to extract total RNA from MSCs, MLE‐12 cells, and lung tissue samples. Following the manufacturer's instructions, the Prime Script RT Master Mix (Takara, China) was utilized to synthesize complementary DNAs (cDNAs). RT qPCR was conducted on a Light Cycler 480 detection system with the iTaq universal SYBR Green Super Mix. Employing GAPDH as the endogenous control, primers were designed using the Primer 5.0 software (http://www.premierbiosoft.com/primerdesign/) and list as follow: F/*Oct4*: AGTTGGCGTGGAGACTTTGC, R/*Oct4*: CAGGGCTTTCATGTCCTGG; F/*Nanog*: GAACGCCTCATCAATGCCTGCA, R/*Nanog*: GAATCAGGGCTGCCTTGAAGAG; F/*Sox2*: AACGGCAGCTACAGCATGATGC, R/*Sox2*: CGAGCTGGTCATGGAGTTGTAC; F/*Hgf*: TATTGCCCTATTTCCCGTTGTGAAG, R/*Hgf*: CCTACTGTTGTTTGTGTTGGAATGC, F/*Gadph*: ACAACTTTGGTATCGTGGAAGG, R/*Gadph*: GCCATCACGCCACAGTTTC. The 2‐ΔΔCt method was utilized to calculate the mRNA expression levels.

### 2.6 Animal Procedures

6–8 weeks male C57BL/6 mice (18–22 g body weight) were purchased from Beijing Shanghai JieSiJie Laboratory Animal Company (Shanghai, China). All mice were housed in a standard animal care room with a 12 h light/dark cycle and had free access to food and water. All experiments and surgical procedures were executed following the criteria of the NIH and authorized by the Medical Ethics Committee of Shanghai Pulmonary Hospital.

The sepsis‐associated lung injury model mice were created by cecal ligation and puncture (CLP) according to previous studies.^[^ ^14^ ^]^ Sham group received the same procedure expect for CLP. The treatment group nebulized PBS corresponding EVs (10^7^ particles diluted in 1 mL PBS) at 2, 6, and 12 h postoperatively, while one of the CLP group nebulized PBS as a negative control. 24 h after operation, the mice were sacrificed to obtain serum, and lung samples for molecular biology experiments and histological observation. Bronchoalveolar lavage was performed by injecting the lungs with 0.9% saline (0.5 mL) through the main bronchus, and this procedure was repeated three times. The bronchoalveolar lavag fluids (BALF) was collected for protein concentrations and cell count determine.

In the survival experiments, animals were subjected to the various treatments as previously described, and their survival status was meticulously documented on a daily basis. The mice were euthanized on day 7 after survival data had been collected.

### 2.7 Lung Histopathology

The lung tissues were preserved using a 4% solution of paraformaldehyde for a duration of 24 h. After that, the preserved lung tissues were embedded in paraffin using established techniques. Following this, the paraffin blocks were sliced into 5 µm thick sections and subjected to hematoxylin and eosin (H&E) staining. The resulting sections were examined using a microscope, and the assessment of lung tissue injury severity was evaluated using the following criteria,^[^ ^15^ ^]^ including intra‐alveolar congestion, intra‐alveolar hemorrhage, presence of neutrophils in air spaces or vessel walls, and the thickness of the alveolar wall/hyaline membrane. A numerical score ranging from 0 to 4 was assigned to each criterion, indicating the level of injury severity, with higher scores representing more severe damage. The total score for each criterion was then calculated as the lung injury score.

After dewaxing and hydration, five µm paraffin sections were blocked with 1% BSA for 15 min in a humid box. The sections were then incubated with the primary antibody at 4 °C overnight to allow for optimal antigen‐antibody interaction. Subsequent to three washes with PBS, the sections were incubated with either FITC or Cy3‐conjugated secondary antibodies in the dark at room temperature for 1 hour to ensure specific signal detection. Nuclear counterstaining was performed with DAPI for 5 min to visualize cell nuclei. The slides were ultimately examined using a fluorescence microscope to capture the immunofluorescence signals.

Terminal deoxynucleotidyl transferase‐mediated dUTP nick‐end‐labeling (TUNEL) assay staining was also performed on paraffin sections using the In Situ Cell Death Detection Kit. A commercial in situ cell death detection kit (Servicebio, China) was used to assay apoptosis in this study according to the manufacturer's protocol. The TUNEL‐positive cells, indicative of apoptotic activity, were enumerated under a fluorescence microscope, providing a quantitative measure of cell death within the tissue sections.

### 2.8 In Vivo Imaging of the EVs

In vivo imaging of EVs was conducted using DiD Perchlorate‐labeled EVs, which were resuspended in PBS for nebulized administration. After an hour post‐administration, the mice were euthanized, and their vital organs were harvested. These organs were then subjected to imaging using the IVIS Lumina imaging system (Xenogen Corporation, Hopkinton, MA, USA) to detect the distribution and accumulation of the labeled EVs. DiD Perchlorate: λem = 646 nm, λex = 663 nm.

### 2.9 Pulmonary Function Test In Mice

Pulmonary function measurements were performed on the FinePointe Series R&C system (EMMS, USA). Prior to the measurements, the animals were administered anesthesia. The mice were intubated and connected with the ventilator for mechanical ventilation to collect ventilation data. Pulmonary function data is analysis for minute volume (MV), dynamic compliance, lung resistance, changes in pleural pressure, and expiratory flow at 50% expired volume.

### 2.10 Western Blotting

Lung tissue and cells were homogenized in ice‐cold PBS to obtain samples containing total proteins. The protein concentration was measured using a BCA Assay Kit (Takara, Japan). Subsequently, the protein samples were separated through electrophoresis and transferred to a polyvinylidene fluoride membrane. The membrane was then treated with a 5% fat‐free milk solution to block any nonspecific binding sites, following which it was incubated with primary antibodies. Following the washing step, a HRP‐conjugated secondary antibody against rabbit or goat IgG (dilution of 1:2000, Bioword, U.S.A.) was introduced and incubated. The immunoreactive bands were then detected using an ECL chemiluminescence kit (Amersham, Arlington Heights, IL). The protein intensity was quantified using Image Lab software and normalized to the internal control protein, β‐Actin or GAPDH. Previous studies have shown that GAPDH is stable during the cell three‐dimension culture.^[^ ^16^ ^]^

### 2.11 Enzyme‐Linked Immunosorbent Assay (ELISA)

The serum concentration of IL‐6, and TNF‐α were assessed by commercial assay kit according to the manufacturer's protocol (Thermo Fisher Scientific; USA).

### 2.12 RNA Sequencing and Data Analysis

Total RNA was extracted from various samples using Trizol reagent (ThermoFisher Scientific, 15 596 026). RNA sequencing and analysis were conducted by Beijing Gene+ (China), with differentially expressed genes identified at *p* ≤ 0.05 and |log_2_FC|≥1. For Kyoto Encyclopedia of Genes and Genomes (KEGG) pathway and Gene Enrichment analysis, a combination of DAVID Functional Annotation Bioinformatics (https://david.ncifcrf.gov/) and CytoScape (http://www.cytoscape.org) was used in our study.

### 2.13 Proteomics and Data Analysis

EVs proteins from the 2D or 3D‐cultured MSCs were collected and further sent to Wuhan Metware Biotechnology for proteomics analysis. Differentially expressed proteins (DEPs) were screened based on the thresholds of *p* ≤ 0.05, and |log_2_FC|≥1. Afterward, the screened DEPs were subjected to multiple bioinformatic analyses, including Gene Ontology (GO) annotation and KEGG pathway enrichment analysis.

### 2.14 Small Interfering RNA (siRNA) Experiments

Cells were seeded at a density of 5 × 10^5^ per well in 12‐well plates and cultured to reach 50% confluence. Target gene knockdown was achieved by transfecting cells with siRNAs and EZ Trans reagents (LIFE iLAB BIO, China), following the manufacturer's protocol. Post‐transfection, cells were incubated at 37 °C with 5% CO_2_ for 48 h. Transfection efficiency was subsequently assessed using quantitative real‐time polymerase chain reaction (qRT‐PCR) analysis. The siRNAs used were synthesized by Sangong Biotech (China); with the sequences are listed as follow: GGAGAUACUACACCUACAATT (5′‐3′), UUGUAGGUGUAGUAUCUCCTT (3′‐5′).

## 3 Result

### 3.1 Scheme of 3D Spheroid Culture and Characterization of 3D‐MSCs

After three serial passages, impurities were removed and the remaining cells were homogeneous, spindle shape, showing good growth activity (Figure 1C). The phenotypic profile of BMSCs was determined by flow cytometry. The results showed that the isolated cells positively expressed CD29, CD49e, and CD90 (over 95% of positive rate), but negatively expressed CD45. (Figure 1D,E). Figure 1A shows the protocol of agarose micro multi‐well dishes. Almost all BMSCs were generated into 3D spheroid after seeding in 3D culture dish for 12 h (Figure 1B). After the stem cell spheres were replanted in Petri dishes for 48 h, the BMSCs in the spheres radiated around the spheres and maintained good vitality (Figure 1C). Flow cytometric results revealed that over 95% of these cells expressed CD29, CD49e and CD90, and were negative for CD45, consistent with the current BMSCs criteria (Figure 1D,E). However, the relative size of cells was decreased to 70%–80% of the original size by flow cytometric forward scatter (FSC) (Figure 1F,G). In fact, the cells in the core of sphere may maintain a smaller volume, which indicates that the cells have structure remodeling during the 3D culture process.

### 3.2 3D‐MSCs Ameliorated Lung Epithelial Cell Injury Model In Vitro

**Figure 2:**
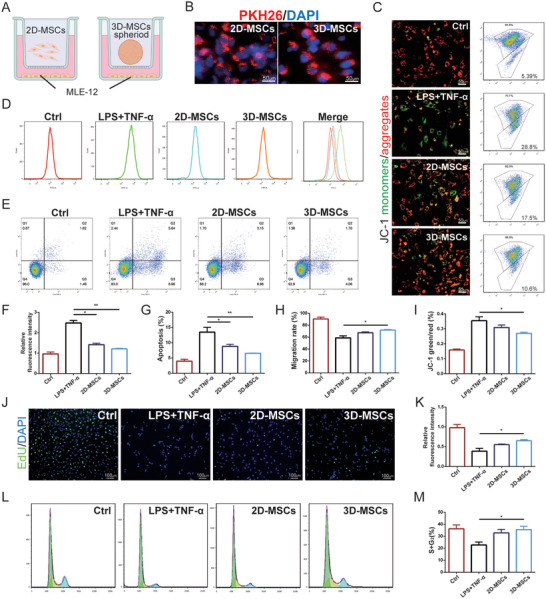
3D‐MSCs alleviated lung epithelial damage in vitro. A) The protocol of 2D or 3D MSC co‐culture with injury lung epithelial cells. B) Uptake of 2D and 3D‐EVs in MLE‐12 cells detected by fluorescence microscope. C,I) MMP assay by fluorescence microscope and flow cytometry (Scale bar: 50 µm). Increases in green fluorescence indicate perturbed MMP. D,F) intracellular ROS assay (count) and quantification of ROS. E,G) Annexin V/PI assay and quantification of apoptosis rate. H) Cell migration analysis. J,K) Cell proliferation was measured using EdU assays and quantification. (Scale bar: 100 µm). L,M)The cell cycle assay was conducted to analyze the proliferation viability. Mean ± SE, *n* = 4. * *P* < 0.05, ** *P* < 0.01.

To investigate the therapeutic efficacy of 3D‐MSCs on lung epithelial injury in sepsis‐induced ALI in vitro, a non‐contact transwell co‐culture system was employed (**Figure** **2**A). PKH26 (red) labeling was utilized to track the delivery of MSC‐derived EVs to recipient cells. Fluorescence imaging revealed the presence of PKH26‐positive spots on MLE‐12 cells, signifying a high uptake efficiency of EVs by the recipient cells (Figure 2B). The loss of mitochondrial membrane potential (MMP) was associated with early apoptosis and mitochondria function. JC‐1 probe was used to analyze the effects of MSCs on the loss of MMP in injured MLE‐12 cells. The results indicated that the 3D‐MSCs played a more significant role in preserving mitochondrial fitness compared to 2D‐MSCs in MLE‐12 cells (Figure 2C,I). Additionally, 3D‐MSCs significantly reduced the levels of reactive oxygen species (ROS) in injured cells (Figure 2D,F). To evaluate the anti‐apoptotic effects of EVs on MLE‐12 cells, an Annexin V/PI assay was conducted. Results demonstrated that the 3D‐MSCs group exhibited superior anti‐apoptotic effects compared to the 2D‐MSCs group (Figure 2E,G). The scratch wound assay demonstrated a significant improvement in the migratory ability of MLE‐12 cells after a 24‐h co‐culture with 3D‐MSCs, compared to the cell model group (Figure S1A, Supporting Information; Figure 2H). EdU staining was employed to assess the influence of MSCs on the proliferation of LPS and TNF‐α induced MLE‐12 cells (Figure 2J). Flow cytometry was used to quantify the EdU‐positive cell ratio, revealing a significant increase in the fluorescence density of EdU‐positive cells in the 3D‐MSCs group relative to the injury model group (Figure S1B, Supporting Information; Figure 2K). Cell cycle assays further substantiated the proliferative effects observed in the 3D‐MSCs group, with 35.5% of cells entering the S and G2 phases, a higher percentage compared to the LPS and TNF group and the 2D‐MSCs group (Figure 2L,M). All these data suggest that 3D‐MSCs exhibit superior therapeutic effects in epithelial injury in vitro, suggesting their potential as an effective treatment for sepsis‐induced ALI.

### 3.3 Integral Analysis of Transcriptome of 3D‐MSCs

**Figure 3:**
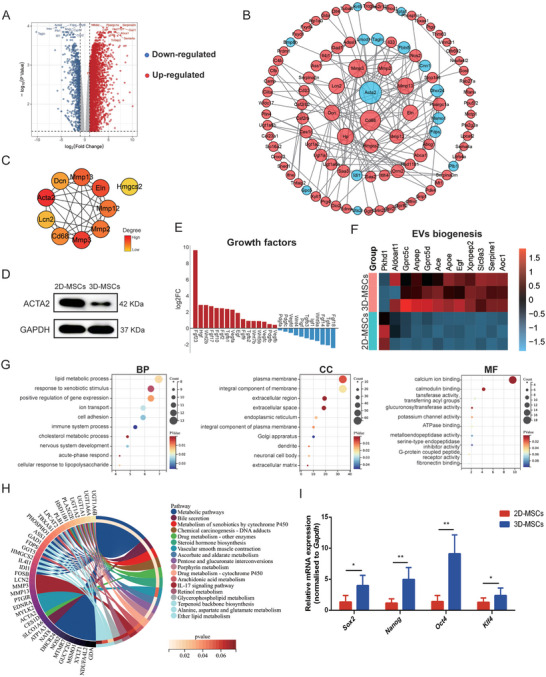
RNA‐sequencing analysis of 2D and 3D‐MSCs. A) Volcano plots of significant DEGs (3D/2D). Red dots represent up‐regulated genes, blue dots represent down‐regulated genes. B) The PPI network of DEGs. C) The top 10 hub genes ranked using MCC plug‐in of Cytoscape. D) The protein expression level of the most significantly downregulated gene *Acta2*. E) Expression cluster of Growth factor family genes. F) Expression heatmap of genes related to EV biogenesis. G) GO enrichment analysis of DEGs. H) KEGG pathway analysis of DEGs. I) Stem cell gene expression. Mean ± SE, *n* = 4. ^*^ *P* < 0.05, ^**^ *P* < 0.01.

To assess the influence of 3D culture on gene expression and to uncover the mechanisms behind the enhanced therapeutic potential of 3D‐MSCs, we performed a high‐throughput RNA‐sequencing analysis of the 3D‐MSCs transcriptome. First, Principal Component Analysis (PCA) was employed to delineate the genetic distinctions between 3D‐MSCs and 2D‐MSCs, as depicted in Figure S2B (Supporting Information), revealing distinct gene expression clusters associated with each culture method. A total of 4795 Differentially Expressed Genes (DEGs) were identified from the expression profile datasets, with a log2 fold change > 1 and an adjusted p‐value < 0.05 (**Figure** **3**A). To visualize the expression patterns of these DEGs, a heatmap was constructed to illustrate the expression matrix of the top 200 DEGs, which included 176 up‐regulated genes and 24 down‐regulated genes (Figure S2C, Supporting Information). To identify hub genes, the top 200 DEGs were subjected to analysis using the STRING database, and a Protein‐Protein Interaction (PPI) network was generated using Cytoscape software (Figure 3B). Subsequently, the top 10 genes with high connectivity scores were identified using the CytoHubba/MCC plugin in Cytoscape, indicating their potential significance in the PPI network or in 3D cell spheroid formation (Figure 3C). The gene with the highest ranking among them is *Acta2*, which plays a role in cell motility, structure, and integrity. It also shows obvious downregulation at the protein level (Figure 3D). A detailed list of these top 10 genes, including their full names and functions, is presented in **Table** **1**. Additionally, the mRNA of growth factors in MSCs cultured in 3D exhibited substantial upregulation (Figure 3E). Genes that positively regulate cell proliferation were also extensively upregulated, potentially linking to their augmented therapeutic potency (Figure S2E, Supporting Information). GO analysis annotated the DEGs and their gene products with respect to biological processes (BP), cellular components (CC), and molecular functions (MF), as shown in Figure 3G. The upregulation of genes associated with EV biogenesis (Figure 3F) and extracellular matrix remodeling (Figure S2D, Supporting Information) implies a potential role for EVs in 3D‐MSC therapy. KEGG pathway analysis indicated that the DEGs were predominantly enriched in metabolic pathways, including “Metabolism of xenobiotics by cytochrome P450,” “Drug metabolism–other enzymes,” and “Ascorbate and aldarate metabolism” (Figure 3H). Furthermore, the expression of stemness‐associated genes *Oct4*, *Sox2*, *Nanog*, and *Klf4* was upregulated in 3D spheroid‐cultured MSCs, suggesting the induction of a more primitive phenotype compared to 2D culture conditions (Figure 3I).

**Table 1:** Ten hub genes and their functions.

Rank	Gene symbol	Description	Function	logFC	adj P
1	Acta2	actin alpha 2, smooth muscle	Encodes one of six different highly conserved actin proteins.	−4.99	1.00E‐4
2	Hmgcs2	3‐hydroxy‐3‐methylglutaryl‐CoA synthase 2	Enables hydroxymethylglutaryl‐CoA synthase activity.	5.34	3.18E‐3
3	Lcn2	lipocalin 2	Enables iron ion binding activity. Involved in innate immune response; positive regulation of cold‐induced thermogenesis; and siderophore transport.	6.89	1.78E‐4
4	Mmp13	matrix metallopeptidase 13	A member of the matrix metalloproteinase family that plays a role in degradation of extracellular matrix proteins	5.45	1.42E‐4
5	Mmp2	matrix metallopeptidase 2	A member of the matrix metalloproteinase family of extracellular matrix‐degrading enzymes	5.34	7.71E‐4
6	Cd68	CD68 antigen‐	Involves in several processes, including cellular response to lipopolysaccharide; cellular response to oxidised low‐density lipoprotein particle stimulus.	4.19	1.52E‐3
7	Mmp3	matrix metallopeptidase 3	A member of the matrix metalloproteinase family of extracellular matrix‐degrading enzymes.	8.92	5.78E‐4
8	Mmp12	matrix metallopeptidase 12	A member of the matrix metalloproteinase family of extracellular matrix‐degrading enzymes.	4.95	2.56E‐3
9	Eln	elastin	Encodes elastin of the extracellular matrix protein	4.48	4.58E‐4
10	Dcn	decorin	Encodes preproprotein is processed to produce a mature protein that regulates collagen fibril assembly in the extracellular space.	5.15	2.96E‐4

### 3.4 Characterization of 3D‐MSCs Derived EVs

**Figure 4:**
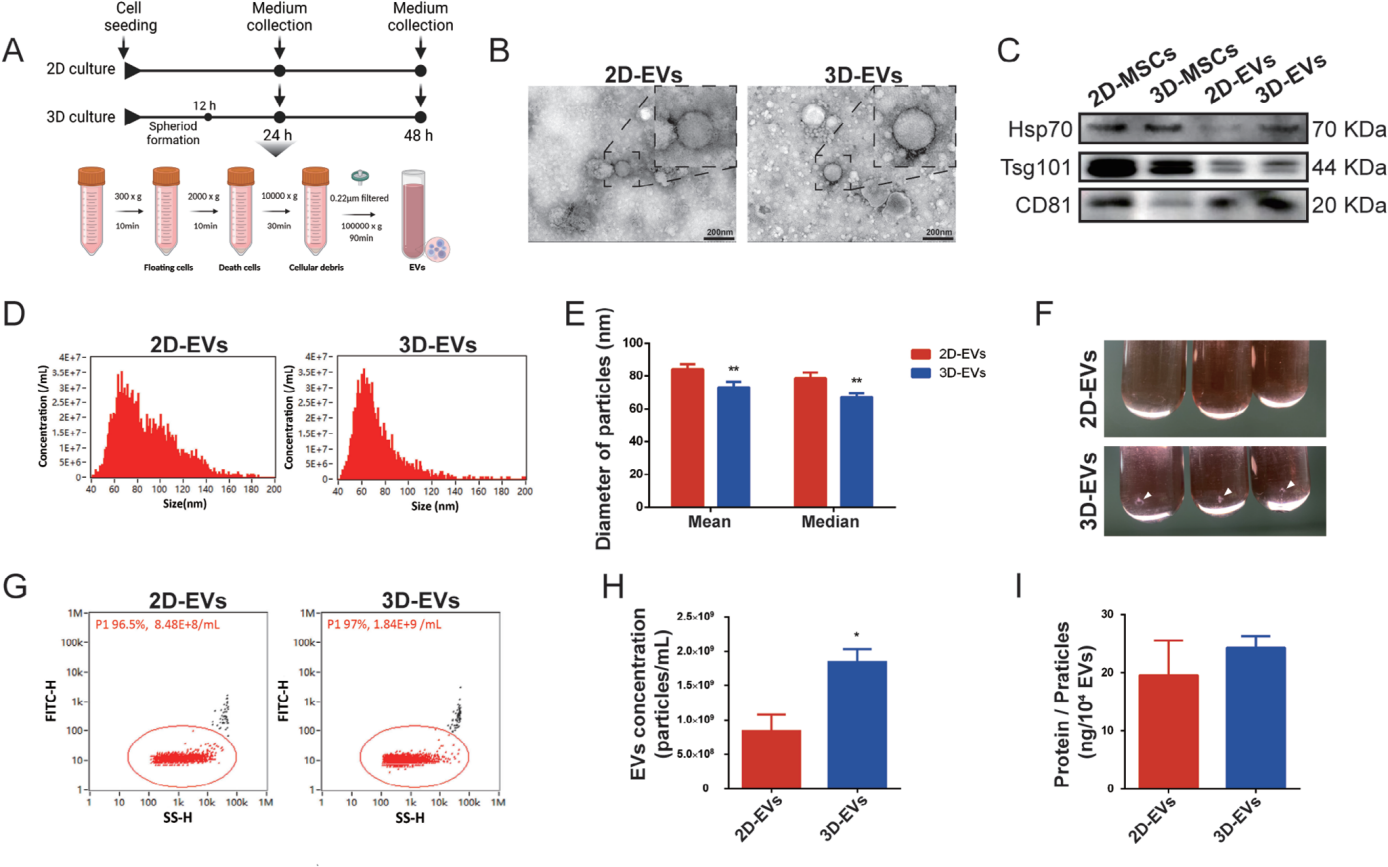
Characterization of EVs derived from 2D and 3D cultured MSCs. A) Protocol of EV isolation from MSCs supernatant. B) The morphology of EVs from 2D and 3D‐MSCs was detected by TEM (Scale bar: 200 nm). C) Protein expression of MSC‐EVs positive markers (Hsp70, TSG101, and CD81). D,E) The particle size distribution of MSC‐EVs was measured by nanoflow cytometer. F) Ultracentrifugation enrichment of 2D and 3D‐EVs. G,H) quantitative analysis of EV production from 2D and 3D culture. I) Total protein quantification in 2D and 3D‐EVs. Mean ± SE, *n* = 4. ^*^ *P* < 0.05, ^**^ *P* < 0.01.

Extensive research has highlighted the therapeutic potential of EVs derived from MSCs through their paracrine signaling capabilities for various conditions.^[^ ^17^ ^]^ Consequently, EVs were isolated via ultrafiltration and ultracentrifugation for further experimental analysis (**Figure** **4**A). Transmission electron microscopy (TEM) images revealed that both 2D‐EVs and 3D‐EVs exhibited the characteristic cup‐base shape (Figure 4B). Western blotting confirmed the presence of specific EV protein markers, including HSP70, TSG101, and CD81, which were enriched in both 2D‐EVs and 3D‐EVs (Figure 4C). Interestingly, the mean and median diameter of the 3D‐EVs (73.08  and 66.97 nm) were significantly smaller than 2D‐EVs (84.31  and 78.57 nm, respectively) (Figure 4D,E). It seems not to be reported in most previous related studies, except the study of Yuan et al.^[^ ^11^ ^]^ The supernatant of quantitative cell count was collected by ultracentrifugation, 3D‐EVs groups showed more particle precipitation (Figure 4F). The result of nanoFCM showed that the number of 3D cultured production was 2‐fold than 2D group (Figure 4G,H), while the EV‐based protein content was no difference between the two groups (Figure 4I). These results indicated that the harvested EVs from both 2D and 3D cultures conform to the typical criteria; however, there exist discrepancies in their properties.

### 3.5 3D‐EVs Ameliorated Sepsis‐Induced ALI Model In Vivo

**Figure 5:**
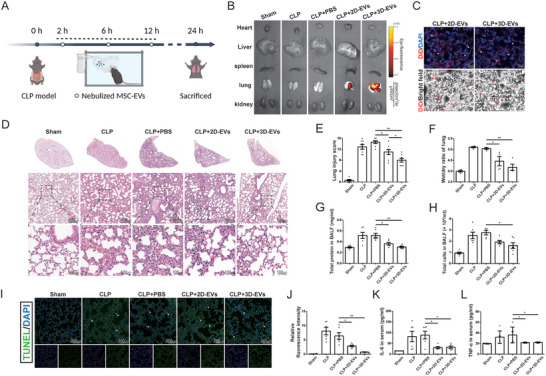
The effects of 3D‐EVs on ameliorating sepsis‐induced acute lung injury A) The schematic diagram of study design and procedure in vivo. B) Fluorescence imaging of tissues from different groups with or without nebulization. C) Detection of DID‐labeled EVs uptake by lung tissues in both fluorescence and bright field (Scale bar: 50 µm). D) Representative images of H&E‐stained lung tissue sections. (Top: Scale bar: 500 µm Bottom: Scale bar: 100 µm). E) The pathological lung injury score was calculated according to the H&E staining. F) Wet‐to‐dry ratio of lung tissues. G) Total protein concentration in BALF. H) Total cell counts in BALF. I), J) TUNEL staining of apoptotic cells and quantitative analysis of fluorescence intensity by ImageJ (Scale bar: 200 µm). Levels of K) IL‐6 and L) TNF‐α in plasma measured by ELISA. Mean ± SE. ^*^ *P* < 0.05, ^**^ *P* < 0.01.

We next investigated the protective effects of 3D‐EVs in the septic ALI model in vivo, and the animal procedure is shown in **Figure** **5**A. First, to demonstrate that EVs could be taken in by lung epithelial tissue in vivo, EVs were labeled with a lipophilic far‐red fluorescent dye DID and administered to mice via inhalation. After an hour, the in vivo tracing showed that the 2D‐EVs and 3D‐EVs groups enriched red fluorescence in lung tissue (Figure 5B), and the red fluorescence was observed in mice lung alveolus by frozen fluorescent sections, indicating the effective uptake of EVs by the lung epithelial tissue (Figure 5C). Histological staining showed that CLP induced acute injury responses, such as interstitial edema, infiltration of inflammatory cells, destruction of alveoli (Figure 5D,E), and lung cells damage (Figure 5I,J). All these symptoms were significantly relieved after EV administration, with particularly noticeable improvements observed in the 3D‐EVs group. Meanwhile, BALF analysis results revealed that administration of 3D‐EVs significantly reduced the total protein concentration and inflammatory cell exudation in BALF (Figure 5G,H), accompanied by reduction of lung wet‐to‐dry ratio (Figure 5F). EVs also reduced the levels of inflammatory (cytokines IL‐6 and TNF‐α) in serum, although no significant difference was observed between the two intervention groups (Figure 5K,L).

### 3.6 3D‐EVs Maintained Lung Epithelial Barrier Integrity via PI3K/AKT Pathway

**Figure 6:**
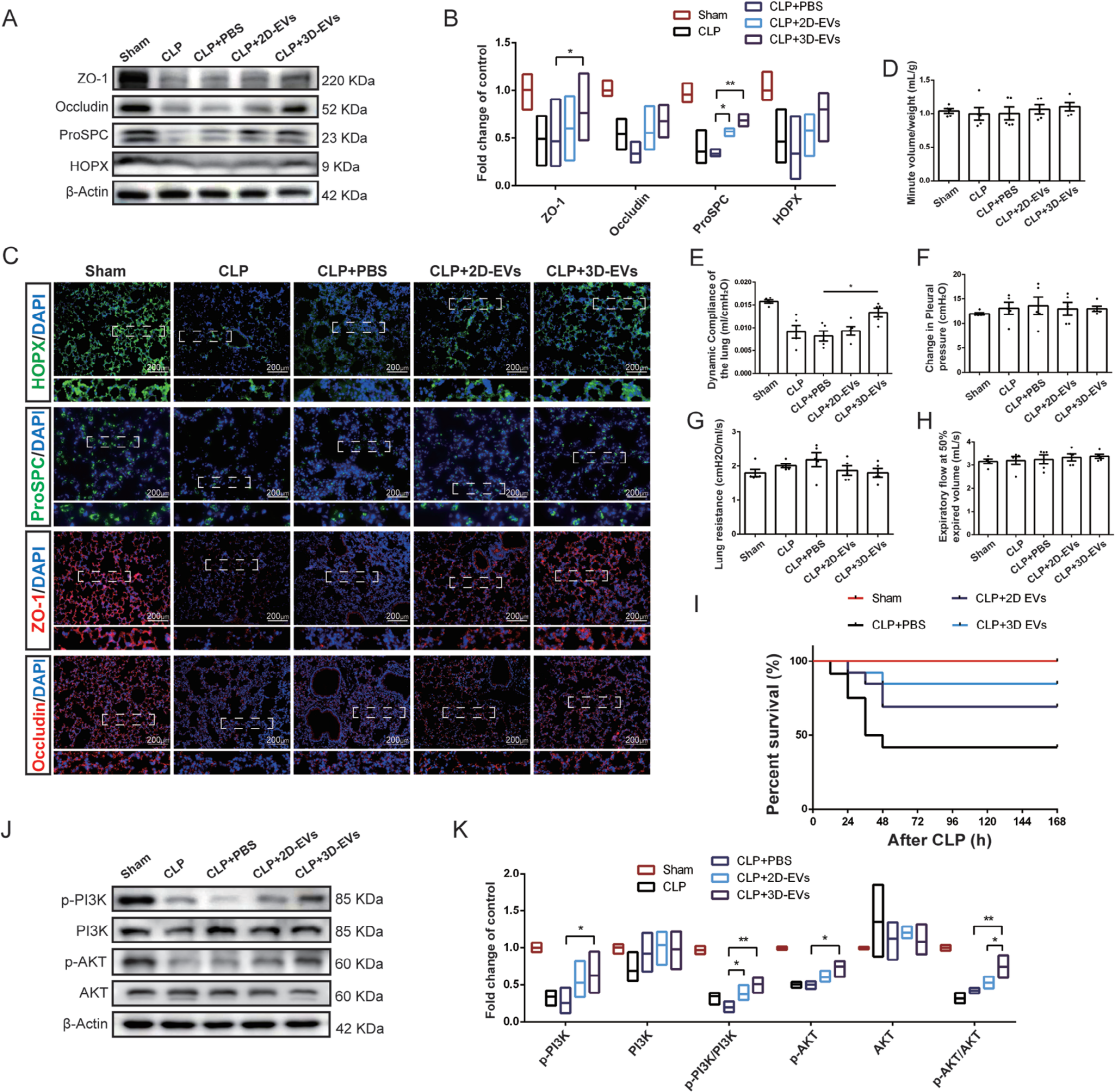
3D‐EVs alleviated sepsis‐induced epithelial barrier damage via PI3K/AKT pathway in vivo. A), B) Expression of ZO‐1, Occludin, ProSPC, and HOPX in lung tissues across different groups, as detected by Western blotting, was quantified using ImageJ and normalized to β‐Actin. C) Immunofluorescence staining showing expression and distribution of ZO‐1 (Red), occludin (Red), ProSPC (Green), and HOPX (Green) in different groups of lung tissues (Scale bar: 200 µm). Quantification of pulmonary function: D) minute volume (MV); E) dynamic compliance; F) lung resistance; G)changes in pleural pressure; H) expiratory flow at 50% expired volume. I) Survival curves of mice in different groups (*n* = 12). J,K) The protein level of PI3K, AKT, PI3K phosphorylation, and AKT phosphorylation was examined by Western blotting. β‐Actin served as a loading control. The relative quantification of the detected signals was determined using ImageJ and normalized to β‐Actin. Mean ± SE. ^*^ *P* < 0.05.

The alveolar epithelium consists of gas‐exchange‐facilitating alveolar type 1 epithelial cells (AT1 cells, HOPX positive) and alveolar type 2 epithelial cells (AT2, ProSPC positive), which are responsible for the secretion of pulmonary surfactants, antioxidants, and other crucial defensive molecules in the lung. Here, we examined the changes in AT1 and AT2 distribution in response to EV treatment post‐CLP injury to assess epithelium damage and rescue (**Figure** **6**A,C). Meanwhile, to demonstrate the effects of 3D‐EVs on the alveolar epithelial barrier, we assayed the expression of ZO‐1 and occludin (Figure 6A,C), which maintain the integrity of the alveolar epithelial barrier. We observed that the protein levels of ZO‐1, occludin, ProSPC, and HOPX declined in pulmonary tissues of CLP model mice (Figure 6B). However, these effects were reversed by inhalation of 3D‐EVs. Additionally, EVs did not significantly alter the relative number of ProSPC‐positive cells (Figure S4, Supporting Information), which may be associated with the early activation of endogenous repair mechanisms in ALI.^[^ ^18^ ^]^ Collectively, 3D‐EVs exhibit superior restorative effects on compromised lung epithelium and epithelial barrier integrity compared to 2D‐EVs.

The integrity of the lung epithelial barrier is crucial for maintaining pulmonary function and preventing pathogenic invasion.^[^ ^19^ ^]^ Subsequent pulmonary function analyses revealed that 3D‐EVs counteracted the decrease in pulmonary compliance induced by ALI, underscoring their therapeutic potential (Figure 6E). Notably, airway resistance parameters in the CLP model were unaltered, and no emphysema was observed. Consequently, other associated indicators displayed negative outcomes (Figure 6D,F–H). The administration of 3D‐EVs to mice with sepsis‐induced ALI also enhanced their survival rates (Figure 6I). The engagement of the PI3K/AKT signaling pathway has been demonstrated to mitigate cellular oxidative stress and to foster cell proliferation, among other beneficial effects.^[^ ^20^ ^]^ By modulating the expression of key genes and proteins involved in these processes, the PI3K/AKT pathway serves as a critical regulator of cellular homeostasis and response to stress.^[^ ^21^ ^]^ Abnormal activation of the canonical PI3K/AKT signaling pathway has been strongly correlated with alveolar destruction, fibrosis, and loss of epithelial barrier integrity. To ascertain whether 3D‐EVs modulate epithelial injury and barrier stability through the PI3K/AKT pathway, we measured the phosphorylation levels of PI3K and AKT in lung protein lysates. Immunoblotting analysis demonstrated reduced expressions of p‐AKT and p‐PI3K in the model group, with a subsequent restoration of these expressions in the lungs of 3D‐EVs treated mice (Figure 6J,K).

### 3.7 3D‐EVs Enriched and Delivered HGF, Maintaining the Integrity of the Lung Epithelial Barrier

**Figure 7:**
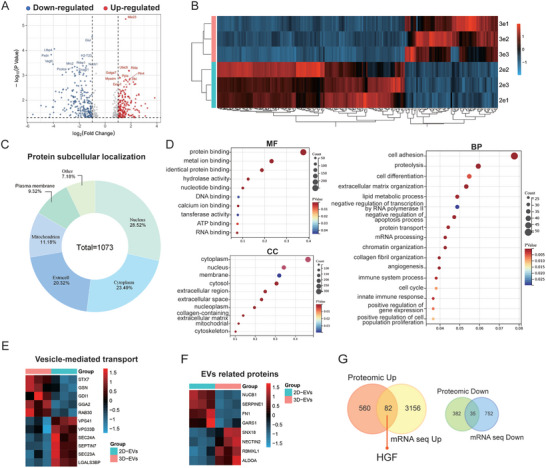
Proteomics analysis of 2D and 3D‐EVs. A) Volcano plots of DEPs (3D‐EVs/2D‐EVs). Red dots represent up‐regulated proteins, blue dots represent down‐regulated proteins. B) Heatmap of top 200 DEPs from proteomics analysis. C) Protein subcellular location of DEPs. D) GO enrichment analysis of DEPs. E,F) Heatmap of EVs‐related proteins. G) Venn diagram of co‐expressed genes and proteins identified in DEGs and DEPs.

Previous RNA‐sequencing studies have revealed a broad upregulation of growth factor gene clusters in 3D‐MSCs. To ascertain if the corresponding EVs encapsulate functional proteins that contribute to therapeutic outcomes, we conducted a proteomics analysis of these EVs. Data normalization via the Robust Multichip Average (RMA) method rendered the distributions clear and stable, with selected values in the violin plot used for subsequent analyses (Figure S3A, Supporting Information). Principal component analysis (PCA) was then employed, revealing a distinct segregation between DEPs from 2D‐EVs and D‐EVs EVs in the 3D score plot (Figure S3B, Supporting Information). The volcano plots of DEPs are depicted in **Figure** **7**A. The heatmap of the top 200 significant DEPs is presented in Figure 7B, with hierarchical clustering analysis demonstrating the clear discrimination between DEPs from 3D‐EVs and 2D‐EVs. In terms of protein subcellular localization, the proteome was predominantly composed of nucleic (28.52%), cytoplasmic (23.48%), and extracellular (20.32%) components (Figure 7C). A total of 1073 DEPs were integrated into a protein‐protein interaction (PPI) network and visualized using Cytoscape (Figure S3C, Supporting Information). KEGG pathway analysis, comparing 3D‐EVs to 2D‐EVs, identified the PI3K/AKT, MAPK, Ras, and Rap1 signaling pathways as being DEP‐enriched (Figure S3D, Supporting Information). GO analysis revealed that 3D‐EVs contained proteins involved in cell adhesion, proteolysis, and extracellular matrix organization (Figure 7D). The differential expression of proteins implicated in vesicle‐mediated transport and EV biogenesis is illustrated in Figure 7E,F, suggesting that 3D culture systematically modifies the EVs derived from MSCs. Furthermore, the Venn diagram displays genes and proteins that are concurrently upregulated or downregulated in the transcriptomics of 3D‐MSCs and proteomics of 3D‐EVs. Notably, there were 82 co‐upregulated and 35 co‐downregulated genes or proteins, with HGF being the only upregulated growth factor in both expression profiles (Figure 7G). Subsequently, Western blotting analysis confirmed that 3D‐EVs contained higher levels of HGF protein compared to 2D‐EVs (Figure S5, Supporting Information).

**Figure 8:**
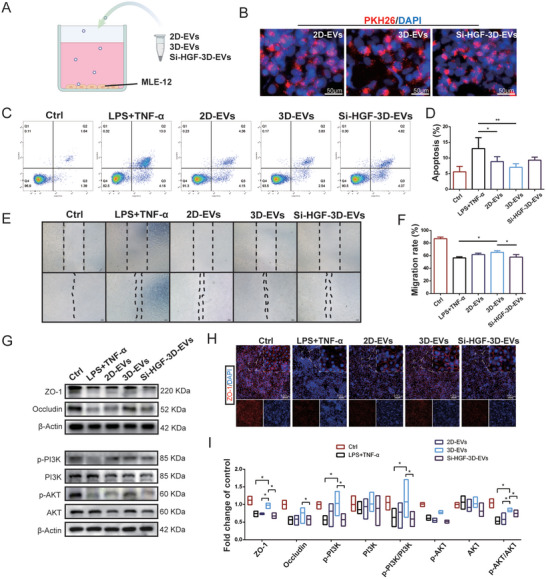
3D‐EVs delivered HGF to protect lung epithelial cells from apoptosis and epithelial barrier damage. A) The protocol of 2D, 3D‐EVs, or Si‐HGF‐3D‐EVs co‐culture with injured lung epithelial cells. B) Uptake of 2D, 3D‐EVs, or Si‐HGF‐3D‐EVs in MLE‐12 cells detected by fluorescence microscope. C), D) Annexin V/PI assay and quantification of apoptosis rate. E,F) A scratch experiment was used to assess cell migration (Scale bar: 200 µm). G,I)The protein expression level of ZO‐1, occludin, PI3K, AKT, PI3K phosphorylation, and AKT phosphorylation was examined by Western blotting. β‐Actin served as a loading control. The relative quantification of the detected signals was determined using ImageJ and normalized to β‐Actin. H) Immunofluorescence staining showing expression and distribution of ZO‐1 (Red) in MLE‐12 cells (Scale bar: 100 µm). Mean ± SE, *n* = 4. ^*^ *P* < 0.05, ^**^ *P* < 0.01.

**Figure 9:**
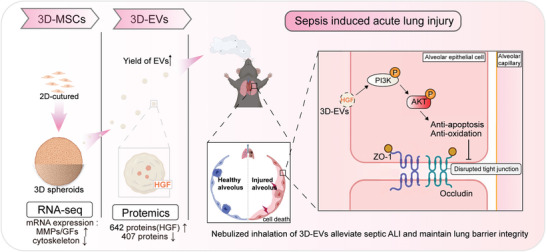
MSCs cultured in 3D conditions up‐regulated the expression of matrix metalloproteinases (MMPs) and growth factors (GFs), and increased the production of extracellular vesicles (EVs). Proteomic analysis showed that 642 proteins were up‐regulated in 3D‐EVs (including HGF). Moreover, 3D‐EVs are enriched with HGF and deliver it to protect lung epithelial cells from apoptosis and barrier damage in septic ALI through the PI3K/AKT pathway. The figure is created by Biorender.com.

Among other growth factors, HGF is a well‐recognized anti‐apoptotic and pro‐angiogenic factor. Generally, cells express Met and respond to HGF by activating PI3K/AKT and ERK1/2 signalling.^[^ ^22^ ^]^ To evaluate the role of HGF in the protective effects of 3D‐EVs against sepsis‐induced ALI and epithelial barrier damage, we performed HGF silencing using siRNA in vitro (**Figure** **8**A). First, to confirm the cellular uptake in vitro, EVs were labeled with a red fluorescent lipid dye PKH26 and incubated with cells. After 24 h, all of the EVs were internalized by cells in the presence of LPS and TNF‐α (Figure 8B). Flow cytometry apoptosis assay demonstrated a significant reduction of the damaging effect of inflammation factors on MLE‐12 cells, an effect that was attenuated in the HGF siRNA group (Si‐HGF‐3D‐EVs) (Figure 8C,D). The scratch wound assay revealed that the migratory capacity of MLE‐12 cells was substantially enhanced after a 24‐hour treatment with 3D‐EVs, as compared to the TNF‐α group. However, the healing of the scratched surface was significantly delayed in the HGF siRNA group (Figure 8E,F). Next, Western blot results revealed that HGF siRNA administration partially blocked the therapeutic effects of 3D‐EVs on the expression of tight junction proteins, ZO‐1, and occludin (Figure 8G). As shown in Figure 8H, 3D‐EVs significantly alleviated tight junction protein damage or discontinuous distribution. While HGF‐Si‐3D‐EVs weakened this effect. To determine whether HGF derived from 3D‐EVs affected epithelial function recovery by activating the PI3K/AKT pathway, phosphorylation level of PI3K, AKT was measured in vitro. Immunoblot analysis indicated that 3D‐EVs reversed the decrease in the injury‐induced p‐AKT/AKT ratio and p‐PI3K/PI3K ratio, while HGF‐Si‐3D‐EVs reduced this effect (Figure 8G,I). These cumulative results indicate that 3D‐EVs can partly reverse sepsis‐induced down‐regulation of the PI3K/AKT pathway in mouse lung by delivering HGF and thus ameliorating lung injury (**Figure** **9**).

## 4 Discussion

Over the past few decades, preclinical studies have highlighted the therapeutic potential of EVs, particularly those derived from MSCs, in tissue regeneration and repair.^[^ ^23^ ^]^ This has motivated researchers to harness EVs, particularly those derived from MSCs, as cell‐free therapeutics for a variety of diseases. Notably, EVs from MSCs have been shown to be as efficacious as their parent cells in promoting tissue regeneration, as evidenced by their therapeutic potential across diverse tissue injury models.^[^ ^17a^ ^]^

Despite some progress, sepsis‐induced ALI remains a significant challenge in critical care, characterized by high mortality and limited treatment options. Our previous studies have shown that MSCs and their exosomes protect against sepsis‐induced ALI.^[^ ^24^ ^]^ However, challenges such as low production yields, variable bioactivity, and impurity issues hinder their broader application.^[^ ^25^ ^]^ Recent insights suggest that environmental shifts in the microenvironments of primary cells can alter EV content. In response, researchers are exploring various 3D culture techniques to enhance EV production and therapeutic efficacy. Preliminary studies have indicated that MSCs cultured in 3D spheroids can reduce collagen deposition, improve pulmonary function, and offer therapeutic benefits in animal models of lung disease.^[^ ^26^ ^]^ However, the impact of 3D‐EVs with specific cargo on septic ALI repair remains underexplored. In this study, we describe a novel, cost‐effective technique for rapid MSC spheroid formation and thorough characterization of the EVs they produce, with the aim of clarifying their therapeutic mechanisms in ALI.

During spheroid formation, MSCs exhibited elevated expression of stemness genes without altering surface markers. Previous studies have suggested the consensus that maintenance of stem cell stemness is highly associated with ageing‐related diseases. Increasing MSCs' stemness has been extensively used to enhance their therapeutic effectiveness in treating various diseases.^[^ ^27^ ^]^ In vitro experiments involving non‐contact co‐culture of MSC spheroids and injured lung epithelial cells demonstrated that aggregated MSCs significantly enhance therapeutic efficacy by mitigating early injury and promoting cell proliferation. Additionally, 3D spheroid MSCs are approximately one‐fourth the size of traditionally cultured MSCs,^[^ ^27b^ ^]^ potentially enhancing their lung migration capabilities. Our research supports this, showing a reduction in size for MSCs undergoing spheroid formation. However, the risk of embolism from direct intravascular injection of cell spheres remains.^[^ ^28^ ^]^ Transcriptomic analysis of 3D‐MSCs revealed a notable enrichment in the matrix metalloproteinases family, crucial for extracellular matrix degradation.^[^ ^29^ ^]^ This, along with the downregulation of cytoskeleton genes and reduced cell size, suggests significant cellular restructuring during spheroid formation. Intriguingly, there is a positive correlation between the quantity of shed vesicles, their lytic enzyme content, and the invasive potential of various cell lines.^[^ ^29^ ^]^ This also confirms that 3D culture reduces MSC size by increasing vesicle excretion, likely mediated by reduced cytoskeleton tension.^[^ ^16^ ^]^ Similar to previous studies, these processes may contribute to increased EVs yield, resulting in smaller and more uniform EVs.^[^ ^11^ ^]^ Furthermore, the internal core cells of the sphere exhibit altered metabolic pathways and increased pro‐regenerative paracrine signaling molecules and immunomodulatory factors, enhancing the therapeutic effects of MSCs.^[^ ^30^ ^]^

The nebulization of EVs has been established as an effective administration route for lung diseases.^[^ ^26^, ^31^ ^]^ EVs, when nebulized, are distributed in the lungs, primarily localizing in pulmonary macrophages and airway epithelial cells.^[^ ^32^ ^]^ Upon reaching the lungs, EVs transfer growth factors and other contents to injured alveoli for therapeutic purposes.^[^ ^33^ ^]^ In this study, the therapeutic effectiveness of 3D‐EVs in lung injury of CLP mice was verified through continuous inhalation therapy. Inhalation of EVs partially restored pulmonary structure and inhibited cell apoptosis, although slight lung wall thickening persisted. Interestingly, the injury score and other parameters were significantly higher in the 2D‐EVs group compared to the 3D‐EVs group. Alveolar epithelial cells, critical for maintaining alveolar defense barrier integrity and regulating inflammation and external irritants,^[^ ^34^ ^]^ are compromised in lung injury, leading to increased alveolar permeability, pulmonary edema, and subsequent pathological alterations.^[^ ^27a^ ^]^ The destruction of the pulmonary barrier often involves the disruption of tight junctions, evidenced by reduced expression of key tight junction markers such as occludin, Claudin‐1, and ZO‐1.^[^ ^35^ ^]^ In this study, 3D‐EVs reversed the down‐regulation of occludin and ZO‐1 protein expression associated with ALI barrier function impairment. The infiltration of cells and total protein in BALF was also decreased. Pulmonary function test analysis demonstrated the efficacy of EVs in resolving pulmonary function, with 3D‐EVs exhibiting superior therapeutic effects compared to 2D‐EVs and being the sole treatment capable of reversing the reduction in dynamic lung compliance induced by ALI. This effect may be attributed to their ability to enhance type II pneumocyte synthesis^[^ ^36^ ^]^ and maintain alveolar structural integrity. The normal synthesis and secretion of surfactant protein C play a critical role in ensuring alveolar stability and proper pulmonary function by reducing alveolar surface tension and promoting alveolar re‐expansion.^[^ ^37^ ^]^

Proteomics has been instrumental in elucidating the molecular mechanisms underlying EV‐mediated lung repair. In this study, 51 proteins were implicated in the PI3K−AKT signaling pathway, known to delay cellular senescence and extend organismal lifespan through diverse cellular processes.^[^ ^38^ ^]^ Several proteins were predicted to regulate the MAPK signaling pathway, central to cell proliferation and survival, suppression of the aging process, and promotion of BMMSC differentiation.^[^ ^39^ ^]^ Consistent with previous studies,^[^ ^11^ ^]^ there was minimal overlap between DEGs and secreted DEPs, suggesting specific sorting mechanisms governing EV cargo. Notably, HGF was the only growth factor significantly upregulated in both the parental transcriptome and EV proteomics data. Previously, HGF has been shown to exhibit anti‐apoptotic and anti‐fibrotic properties through the activation of the PI3K/AKT/mTOR pathway, contributing to lung barrier restoration in ALI.^[^ ^35^, ^40^ ^]^ Okouchi et al. found that enhancing endogenous HGF levels in the lungs specifically inhibits alveolar epithelial cell apoptosis, mitigating inflammation and fibrosis following bleomycin‐induced injury.^[^ ^41^ ^]^ Thus, we posit that HGF derived from 3D‐EVs contributes to their therapeutic effects. Furthermore, we discovered that Si‐HGF‐3D‐EVs diminished the protective effects of 3D‐EVs on maintaining lung epithelium barrier integrity. Therefore, 3D‐EVs enriched with and delivering HGF present an effective approach for alveolus epithelial injury treatment.

However, there are several potential limitations to the current study. First, we did not conduct parallel controlled trials between 3D‐MSCs and derived EVs to compare their therapeutic effects. Future research in this direction will aid in selecting cell therapy approaches with or without cells under various disease conditions. Second, only one dose of EVs was administered in this study, guided by previous experience. A dose‐response study to establish the optimal dosage would be beneficial for future pre‐clinical and clinical research. Third, while our data indicate that 3D‐EVs can effectively ameliorate sepsis‐ALI via HGF delivery, further investigation is needed to reveal the specific mechanisms and identify alternative substances playing therapeutic roles in EVs.

In summary, our study demonstrates that 3D culture‐derived MSCs and their EVs enhance anti‐injury ability and maintain alveolar epithelial barrier function in experimental sepsis‐induced ALI. Compared to conventional 2D culture conditions, 3D spheroid culture has been shown to enhance EV yield. Furthermore, 3D‐EVs have the potential to enrich and deliver HGF to injured tissues, activating the PI3K/AKT signaling pathway to exert beneficial effects. Given the high morbidity and mortality rates associated with ALI and the current lack of effective therapies, these findings offer promising prospects for the development of ALI treatments.

## Conflict of Interest

The authors declare no conflict of interest.

## Supporting information



Supporting Information

## Data Availability

The data that support the findings of this study are available from the corresponding author upon reasonable request.
