# Supplementary material for: Engineering Extracellular Vesicles Derived from 3D Cultivation of BMSCs Enriched with HGF Ameliorate Sepsis‐Induced Lung Epithelial Barrier Damage
Source: Adv Sci (Weinh). 2025 Mar 5;12(16):2500637. doi: 10.1002/advs.202500637 (PMC12021063; doi:10.1002/advs.202500637)
Supplement: Supplementary file 1 — Supporting Information [file ADVS-12-2500637-s001.docx]

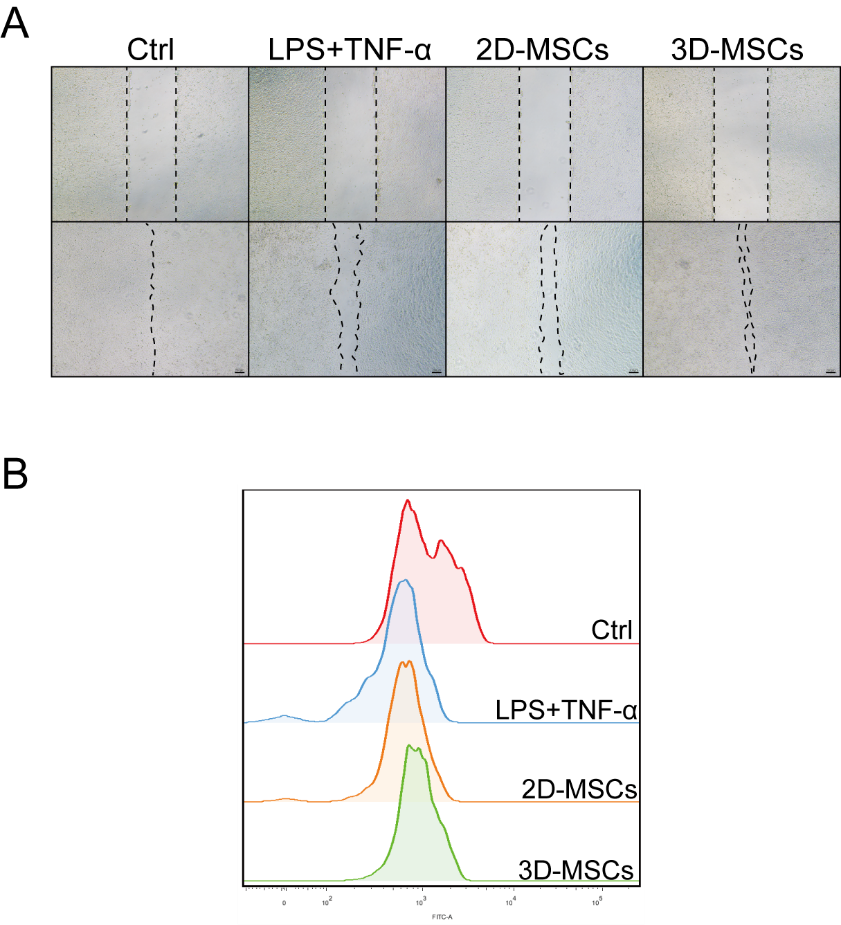


**Figure S1**

(A) A scratch experiment was used to assess cell migration (Scale bar: 200 μm). (B) Cell proliferation was measured using EdU assays and quantification by flowcytometry.


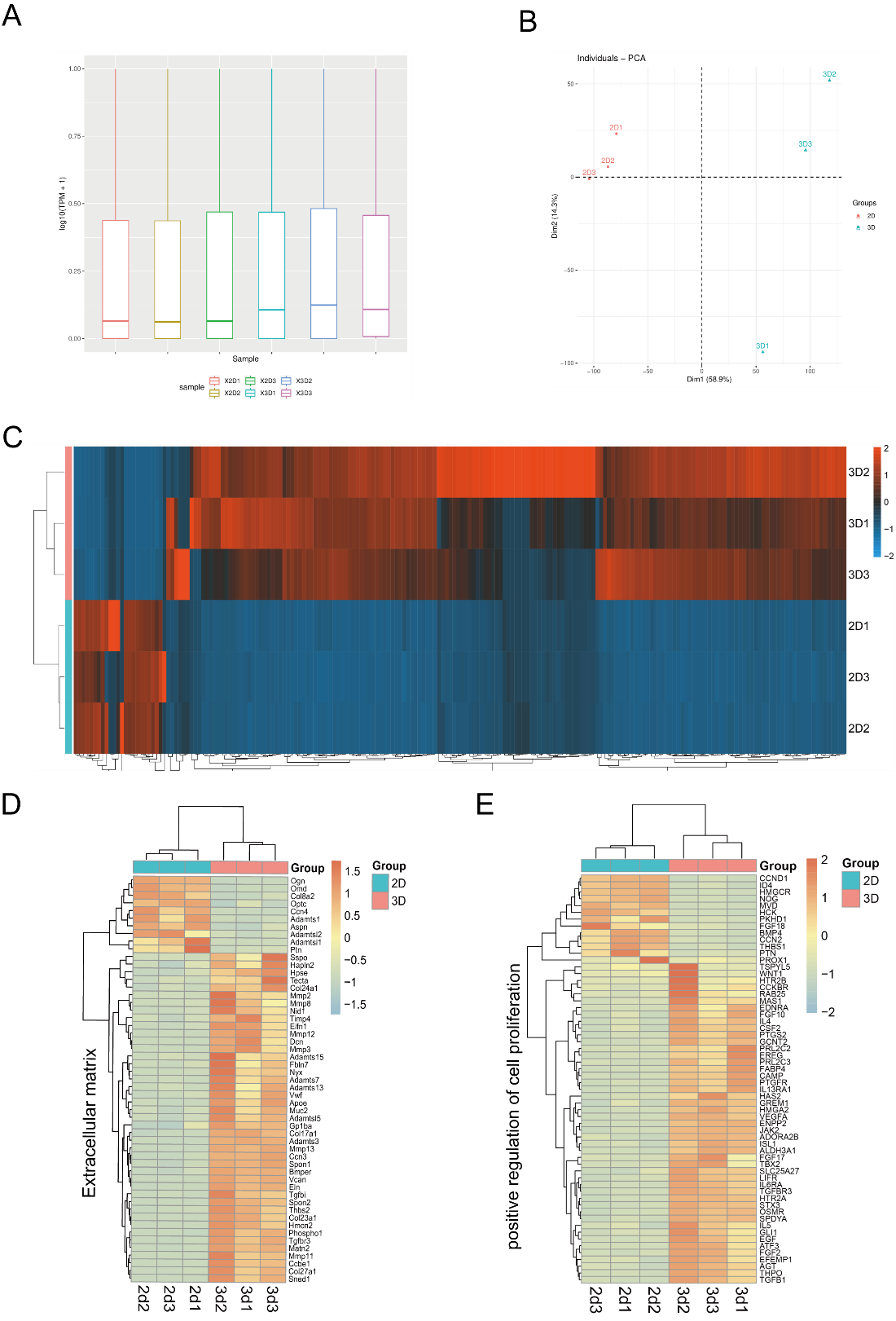


**Figure S2**

(A) Box plots compare the overall expression levels of different samples. (B) PCA analysis. (C) Heatmap of top 200 DEGs from mRNA sequencing. (D) Expression heatmap of genes related to extracellular matrix. (E) Expression heatmap of genes related to the positive regulation of cell proliferation.


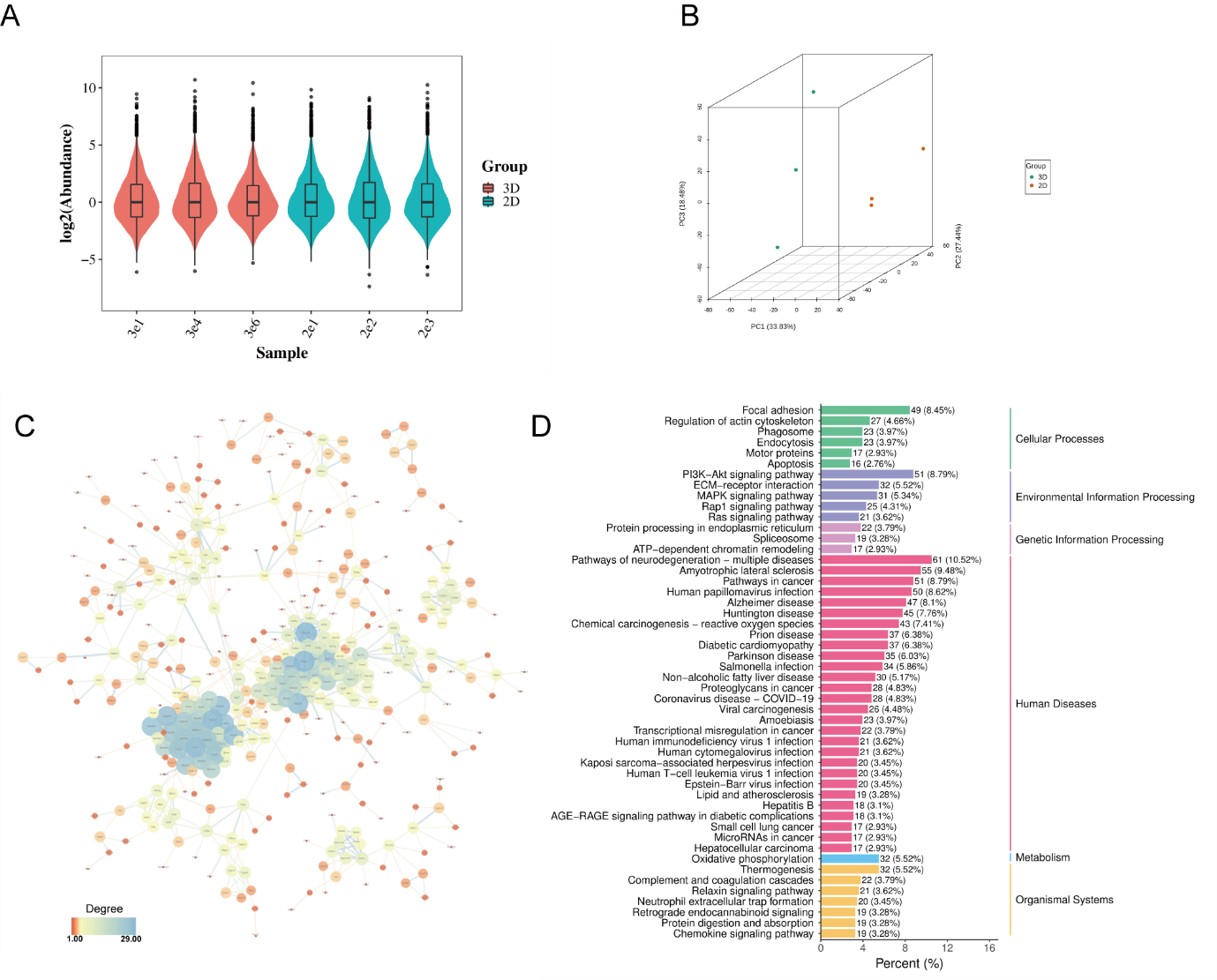


**Figure S3**

(A) Box plots combined with violin plots can reflect the intra-group consistency of biological samples. (B) 3D PCA analysis. (C) The PPI network of DEPs. (D) KEGG pathway analysis of DEPs.


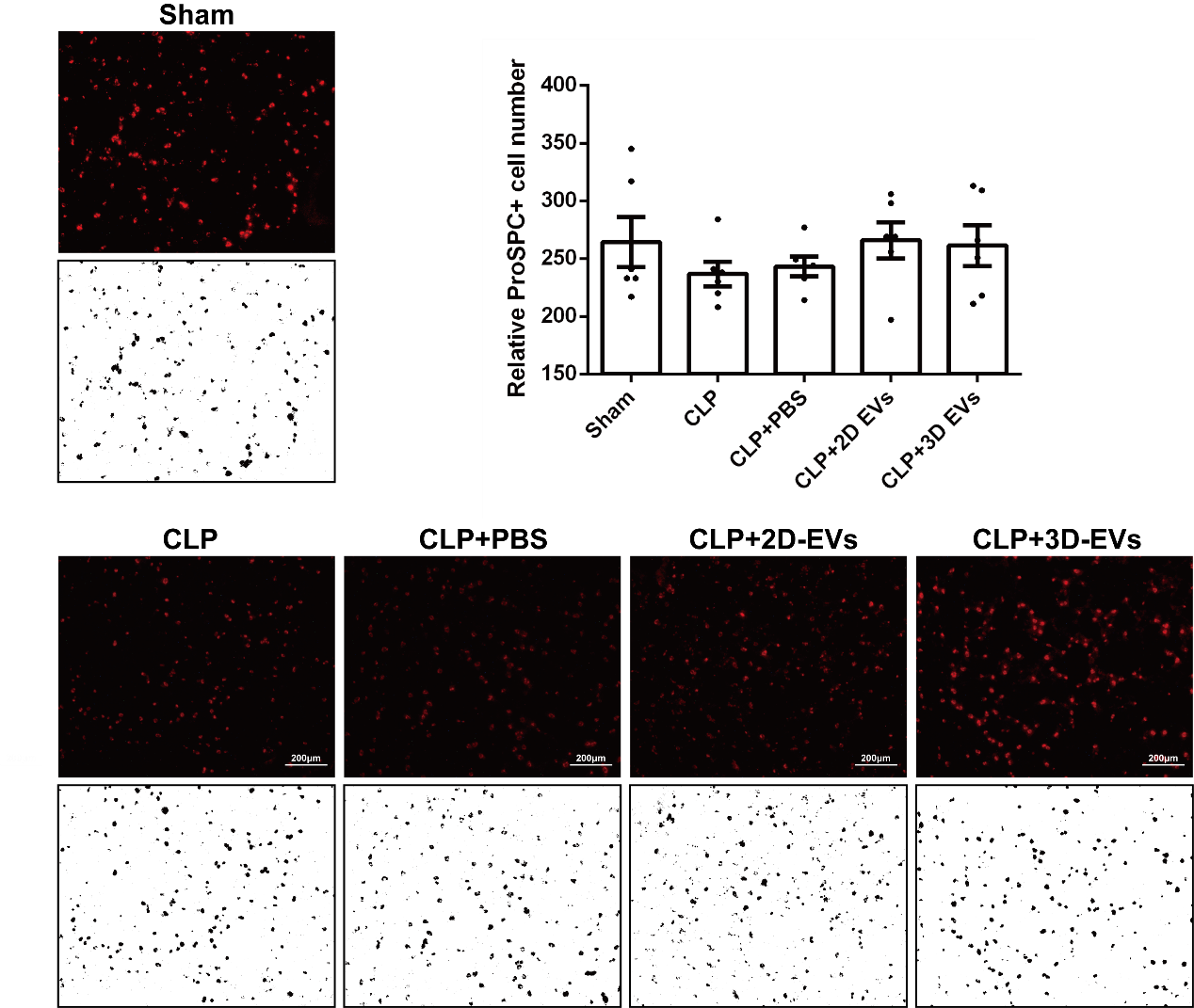


**Figure S4**

Relative count of ProSPC-positive cells (Red).


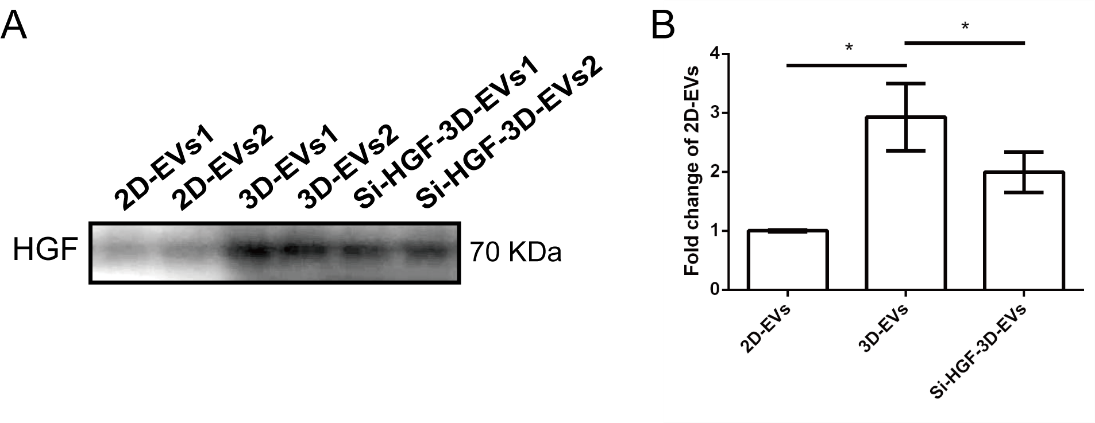


F**igure S5**

(A), (B) HGF protein levels in 2D-EVs, 3D-EVs, and Si-HGF-3D-EVs detected by Western blotting. Total protein quantified by BCA Assay.

**Supplementary Table S1. Antibody information**

| **Antibody** | **Species** | **Vendor (City, State, catalogue)** | **Dilution** | |
| --- | --- | --- | --- | --- |
|  |  |  | **WB** | **HS** |
| ACTB | rabbit | Servicebio (Wuhan, China) | 1:1000 | ND |
| GAPDH | rabbit | Servicebio (Wuhan, China) | 1:1000 | ND |
| ACTA2 | rabbit | Servicebio (Wuhan, China) | 1:1000 | ND |
| Hsp70 | rabbit | Servicebio (Wuhan, China) | 1:1000 | ND |
| Tsg101 | rabbit | diagbio (Hangzhou, China) | 1:1000 | ND |
| CD81 | rabbit | diagbio (Hangzhou, China) | 1:1000 | ND |
| ZO-1 | rabbit | Invitrogen, (Carlsbad, U.S.A) | 1:1000 | 1: 500 |
| Occludin | mouse | Invitrogen (Carlsbad, U.S.A) | 1:1000 | 1: 500 |
| HOPX | mouse | Santa Cruz (Dallas, U.S.A.) | 1:1000 | 1: 500 |
| ProSPC | rabbit | Abcam (San Francisco, CA) | 1:2000 | 1: 1000 |
| pPI3K | rabbit | Cell Signaling Technology (Danvers, MA) | 1:1000 | ND |
| PI3K | rabbit | Cell Signaling Technology (Danvers, MA) | 1:1000 | ND |
| pAKT | rabbit | Cell Signaling Technology (Danvers, MA) | 1:1000 | ND |
| AKT | rabbit | Cell Signaling Technology (Danvers, MA) | 1:1000 | ND |

ND = Not detected; WB = Western blot; HS = Histochemical staining.
